# Stochasticity of flow through microcirculation as a regulator of oxygen delivery

**DOI:** 10.1186/1742-4682-7-29

**Published:** 2010-07-09

**Authors:** Viktor V Kislukhin

**Affiliations:** 1 Transonic Systems Inc, Ithaca, NY, USA

## Abstract

**Objective:**

Observations of microcirculation reveal that the blood flow is subject to interruptions and resumptions. Accepting that blood randomly stops and resumes, one can show that the randomness could be a powerful means to match oxygen delivery with oxygen demand.

**Method:**

The ability of the randomness to regulate oxygen delivery is based on two suppositions: (a) the probability for flow to stop does not depend on the time of uninterrupted flow, thus the number of interruptions of flow follows a Poisson distribution; (b) the probability to resume the flow does not depend on the time for flow being interrupted; meaning that time spent by erythrocytes at rest follows an exponential distribution. Thus the distribution of the time to pass an organ is a compound Poisson distribution. The Laplace transform of the given distribution gives the fraction of oxygen that passes the organ.

**Result:**

Oxygen delivery to the tissues directly depends on characteristics of the irregularity of the flow through microcirculation.

**Conclusion:**

By variation of vasomotion activity it is possible to change delivery of oxygen to a tissue by up to 8 times.

## Introduction

The irregular pattern of blood flow through the microcirculation has been described for all organs and tissues of the body [1]. Estimations of the irregularity of flow by the spectral analysis of a Laser-Doppler signal established that low level of fluctuations in flow is a sign of the pathology [2], particularly of the low level of oxygen consumption [3].

We hypothesized that the consideration of irregularities as a stochastic process reveals the ability of randomness be a regulator of oxygen delivery. The aim of the paper is to test this hypothesis. To reveal the ability of vasomotion to be a regulator of oxygen delivery we start with two well-established facts (a) any organ at rest has only a fraction of microvessels perfused [4], (b) there are about a hundred agents influencing the vasomotor fibers when changing flow from a total stop to a maximum. As a consequence, we can assume that the state of microvessels (open or close) is governed by random causes (a summation of all influences). If a stochastic approach is accepted, then it is reasonable to start with a model based on the assumption that the state of the microvessels depends on the current influences and not previous effects, mathematically speaking, we accept a markovian property for the microvascular system). Main result of the paper can be formulated as follows the less blood flow through an organ, the more effective vasomotion regulation could be.

## Stochastic description of microcirculation

We start with the simple stochastic model: Let T be the time needed for any erythrocytes (RBC) to pass an organ if the RBC is in the move, and T is constant for given organ. The total time to pass an organ by a RBC will be denoted as t, and t - T is the time spent in the interruptions of flow. To find the t let assume that a probability of flow to stop does not depend on the time of uninterrupted flow. Given assumption leads to a Poisson distribution of the number of the interruptions of flow [5] with probability to have, during time T, n interruptions (p_n_) given by:(2.1)

where β is the measure of the intensity of the interruption, and β equals to the mean number of interruptions during 1 sec.

Let also assume that the probability to resume the flow does not depend on the time for the flow being interrupted; meaning that the time τ to resume the flow (after stopping) follows an exponential distribution [5]:(2.2)

where μ is the measure of the intensity to resume the flow and 1/μ is the mean time for resuming of flow.

Since the time t-T is the time spent in n interruptions of flow, then the sum of n independent random variables with distribution given by (2.2), has the distribution:(2.3)

Combination of (2.1) and (2.3) leads to the distribution of the time to pass an organ, P(t):(2.4)

We assume also that the flux of oxygen into tissue, along any microvessels, follows an equation of first order [6].(2.5)

with λ as the intensity of oxygen consumption. Thus the content of oxygen in blood drops from 1 to exp(-λt) if the time t is the time of transition along the given path. Since the time to pass an organ has the distribution (2.4) then fraction of oxygen that passes the organ is integral of exp(-λt) with respect to P(t):(2.6)

One can see in (2.6) Laplace transform of the distribution P(t), and for P(t), as (2.4), Laplace transform is well known [5]:(2.7)

Estimation of the consumed fraction is given by:(2.8)

Equation (2.4), and the consequence of (2.4), equation (2.8) are obtained under supposition that capillaries system is the system of homogenous parallel pathways (time T is constant). However, this is simplification since we have two additional sources of heterogeneity of flow, besides interruption-resuming flow. They are (a) the tortuosity of microvessels, and (b) induced by different causes (local viscosity, tone of vessels, pressures) the changes of flow in any given capillary with time. As the consequence of these heterogeneities of flow, the time T to pass microcirculation if no interruptions happen becomes the variable. If T has a distribution {B(T)}, then the randomization of (2.7) by B(T) will transform (2.8) into the equation with T as mean transit time to pass organ without interruption, T_mean _[5], and the estimation of the consumed fraction is given by:(2.8a)

Thus heterogeneity of flow does not exclude the influence parameters of interruption-resuming on the oxygen consumption.

## Result

The (2.8) reveals that both stochastic characteristics, β and μ, are included into the expression for oxygen consumption. To estimate possible influence of β and μ we should have the ranges of the variation for all variables of (2.8). It should be noticed that parameters of interest (λ, β and μ) have the dimension as [sec^-1^] = [Hz].

Let estimate λ, the intensity of oxygen consumption. It is known [7] that 100 ml of arterial blood contains approximately 18 ml of oxygen. During the time of one second the body consumes, on average, 3 ml of oxygen. Thus, for λT the estimation is 3/18 = 0.17, with T about 5 sec (range 2-7 sec) [8] the λ is about 0.04 (range 0.02-0.08).

Intensity of irregularities estimated by the spectrum of vasomotion activity is from 0 to 2 Hz [9] thus the estimation for the range of and is from 0 to 2.

Introduced stochastic characteristics of microcirculation determinate also the fraction of open microvessels, n_o_. Under a steady state condition, the fraction of microvessels with interrupted flow should correspond to the fraction of vessels with resuming flow, or(3.1)

Thus the expected fraction of open microvessels is:(3.2)

For muscles at rest, n_o _is about 0.05 - 0.1 [7], if n_o _is a constant then one can see from (2.8) that variation of μ and β from small values to the higher values will vary the oxygen consumption about 8-fold (from λT to about 8λT).

## Discussion

Krogh presented the first mathematical model of oxygen consumption by muscular tissue [10]. His model is based on homogeneous presentation of microvascular system, and is known as the Krogh-Erlang cylinder model. Krogh suggested that main mechanism to respond on the increase of the demand of oxygen is the recruitment of microvessels, and thus the increase of blood flow. Homogeneous model of Krogh is the significant simplification of reality. Direct observations of PO_2 _in the blood leaving capillary system reveal heterogeneity of PO_2 _that is impossible for homogeneous flow [11]. An attempt to introduce heterogeneity into description of microcirculation was presented by Pittman as he considered the spatial distribution of microvessels to follow a Poisson distribution [11]. Another approach to introduce heterogeneity of blood flow was presented by Kendal. He introduced compound Poisson-gamma distribution, similar to (2.4), and successfully used it for the description of the self-similarity of microcirculation structure [12].

Another Krogh's assumption that recruitment of microvessels is the leading response of tissue if demand for nutrients is up is well established [4,10], meaning that significant part of microvessels for any organ is out of perfusion.

Model presented in given paper is based on two assumptions (a) the interruption and resuming of flow is Markov process and (b) the probability of more then one interruption/resuming for short period of time is negligible.

Main result (2.8a) is the observation that about 8 fold variation of the demand of oxygen can be satisfied without variation of blood flow by only the changes in intensity of vasomotion. Additionally, the increase of fraction of open microvessels given by (3.2) is result of two independent stochastic processes. The n_o _could be increased if intensity of interruption of flow (β) is down, and also the n_o _could be increased by increase of intensity to resume the flow (μ), thus the recruitment of microvessels becomes complex process.

In publications [13-15] was established that the irregularity of flow due to vasomotion, from periodic to stochastic could be a regulator of oxygen consumption. However, in [13,15] is investigated how oscillation of flow around mean value could influence oxygen consumption. Such, possible, influence on consumption is not considered in given paper. Publication [14] is based on unrealistic assumption of homogeneous perfusion and discrete time. The application of Laplace transform to the distribution of the time to pass an organ, presented in given manuscript, reveals the sensitivity of oxygen consumption to the irregularity of blood flow.

## Conclusion

1. Irregularities of blood flow within microcirculation considered as an stochastic process lead to an compound Poisson distribution for the time to pass an organ.

2. Laplace transform of the time to pass an organ reveals the sensitivity of oxygen consumption to the irregularity of blood flow.

3. By variation of vasomotion activity it is possible to change delivery of oxygen to tissue by up to 8 times.

## Competing interests

The author declares that they have no competing interests.
